# Yeast One-Hybrid Gγ Recruitment System for Identification of Protein Lipidation Motifs

**DOI:** 10.1371/journal.pone.0070100

**Published:** 2013-07-26

**Authors:** Nobuo Fukuda, Motomichi Doi, Shinya Honda

**Affiliations:** Biomedical Research Institute, National Institute of Advanced Industrial Science and Technology (AIST), Higashi, Tsukuba, Ibaraki, Japan; University of Geneva, Switzerland

## Abstract

Fatty acids and isoprenoids can be covalently attached to a variety of proteins. These lipid modifications regulate protein structure, localization and function. Here, we describe a yeast one-hybrid approach based on the Gγ recruitment system that is useful for identifying sequence motifs those influence lipid modification to recruit proteins to the plasma membrane. Our approach facilitates the isolation of yeast cells expressing lipid-modified proteins via a simple and easy growth selection assay utilizing G-protein signaling that induces diploid formation. In the current study, we selected the N-terminal sequence of Gα subunits as a model case to investigate dual lipid modification, *i.e.,* myristoylation and palmitoylation, a modification that is widely conserved from yeast to higher eukaryotes. Our results suggest that both lipid modifications are required for restoration of G-protein signaling. Although we could not differentiate between myristoylation and palmitoylation, N-terminal position 7 and 8 play some critical role. Moreover, we tested the preference for specific amino-acid residues at position 7 and 8 using library-based screening. This new approach will be useful to explore protein-lipid associations and to determine the corresponding sequence motifs.

## Introduction

A wide range of proteins are modified by covalent attachment of fatty acids and/or isoprenoid groups, modifications that play major roles in regulating protein structure and function [1]. Moreover, aberrant expression of lipidated proteins or their biosynthetic enzymes is associated with many diseases, ranging from cancer to neurological disorders [2]. Because recruitment of lipidated proteins to the plasma membrane influences complex signaling pathways that regulate specific physiological functions, there is a great interest in gaining a better understanding of protein trafficking via lipid modification.

Dual lipidation of proteins, which consist of myristoylation and palmitoylation at N-terminal residues, a posttranslational modification that has been conserved from yeast to humans, leads to transport of the modified proteins toward plasma membrane. For some lipid modification, the consensus motifs have been identified. For example, after removal of the N-terminal methionine residue by methionine aminopeptidase, myristate is attached to the N-terminal glycine of protein substrates with the consensus motif Met_1_-Gly_2_-Xaa_3_-Xaa_4_-Xaa_5_-Ser/Thr_6_-Xaa_7_-Xaa_8_ (where Xaa indicates any amino acid residue) [3] in a reaction catalyzed by myristoyl-CoA:protein N-myristoyltransferase (NMT) [4]. Further refinement of this consensus might be possible, however, as it has been shown that not all amino acid residues are allowable at the positions indicated by Xaa [4]–[5]. Cysteine residues within proteins can be acylated with the 16-carbon fatty acid palmitate. Although protein acyltransferases (PATs) have a common DHHC Cys-rich domain [6], the consensus motif for palmitoylation of proteins is still unclear, due at least in part to the diversity of substrates that can be recognized by multiple PATs, including 7 DHHC proteins in yeast and 23 in humans [7].

We previously developed the Gγ recruitment system (GRS), which uses yeast G-protein signaling (pheromone signaling) to detect protein–protein interactions [8]–[10]. This system is based on the observation that transduction of the signal requires localization of the γ subunit of G-proteins (Gγ) to the inner leaflet of the plasma membrane [11]. Deletion of lipidation sites in yeast Gγ (Gγ_cyto_) completely disrupts G-protein signaling [8]; however, protein–protein interactions between a Gγ_cyto_-fused target and a membrane-bound binding partner can restore of G-protein signaling.

**Figure 1:**
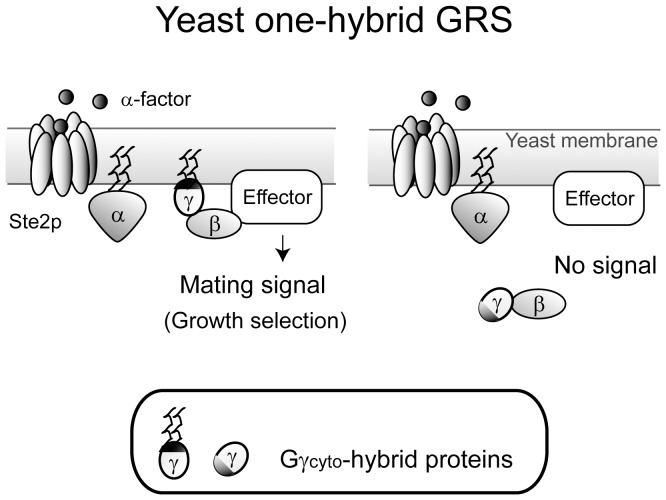
New approach to investigate membrane associations of proteins utilizing the yeast G-protein signal transduction. Wild-type Gγ is lipid-modified at its C-terminus, and localized at plasma membrane to transmit the intracellular signal. An engineered Gγ lacking membrane association (Gγ_cyto_) is fused to the target protein domain or peptide motif, yielding a Gγ_cyto_ hybrid protein. When the target protein domain or peptide motif does not confer membrane association, G-protein signaling is not restored. In contrast, when a Gγ_cyto_ hybrid protein confers plasma membrane localization, G-protein signaling is restored, leading to induction of the mating response and generation of diploid cells.

Here we suggest a new approach to the investigation of protein-lipid associations, yeast one-hybrid GRS (Fig. 1). If a Gγ_cyto_-fused hybrid protein is localized to the plasma membrane, G-protein signaling is recovered, inducing a mating response. Hybrid proteins positive in the assay can then be detected by diploid growth selection [9]–[10]. Thus, using this system, it is possible to ask if a domain fused to Gγ_cyto_ associates with lipid molecules that localize the protein to the plasma membrane.

In the current study, we attached short signal sequences, from 6 to 10 amino acid residues (AA), derived from G-protein α (Gα) subunits to N-terminus of Gγ_cyto_ to append dual lipid modifications, *i.e.,* myristoylation and palmitoylation, resulting in trafficking to the plasma membrane. Gα subunits play a key role in signal transduction that is mediated by lipid modification. It has been reported that lipidation-defective Gpa1 mutants (G2A and C3A) have lost their intrinsic ability to localize to membranes [12]–[13]. Among human Gα proteins, most subunits in the G_i_α subfamily receive both myristoylation and palmitoylation at N-terminal residues, similar to yeast Gpa1. Indeed, human G_i2_α contains the same sequence at N-terminal 6 AA (Met_1_-Gly_2_-Cys_3_-Thr_4_-Val_5_-Ser_6_) as yeast Gpa1 [14]–[15]. We have evaluated the membrane-targeting ability of several N-terminal short sequences thought to receive myristoylation and palmitoylation using yeast one-hybrid GRS.

## Materials and Methods

### Strains and Media

Detailed information about *Saccharomyces cerevisiae* strains BY4741 [16] and MCF4741 [17], as well as about other strains used in this study, including genotypes, are shown in Table 1. Yeast cells were grown in YPD medium containing 1% yeast extract, 2% peptone and 2% glucose, or in SD medium without histidine (SD – His) containing 0.67% yeast nitrogen base without amino acids (Becton Dickinson and Company, Franklin Lakes, NJ, USA), 2% glucose, 20 mg/l uracil, 30 mg/l leucine and 20 mg/l methionine. In either case, 2% agar was added to make solid media (plates).

**Table 1:** Yeast strains and plasmids used in this study.

Name	Description	Reference source
*Yeast strains*		
BY4741	*MAT* **a** *his3*Δ*1 ura3*Δ*0 leu2*Δ*0 met15*Δ*0*	Brachmann et al. [16]
MCF4741	BY4741 *fig1*:: *FIG1-EGFP*-*loxP*-*kanMX4-loxP*	Fukuda et al. [17]
MCF-B1	BY4741 *fig1*:: *FIG1-EGFP*-*loxP*	Present study
MCF-B1L	MCF-B1 *ste18*Δ:: *LEU2*	Present study
BY4742	*MAT* **α** *his3*Δ*1 ura3*Δ*0 leu2*Δ*0 lys2*Δ*0*	Brachmann et al. [16]
BY4743	*MAT* **a** */* **α** *his3Δ1/his3Δ1 leu2Δ0/leu2Δ0 LYS2/lys2Δ0 met15Δ0/MET15 ura3Δ0/ura3Δ0*	Brachmann et al. [16]
*Plasmids*		
pHY-2GA	*2* µ ori, *HIS3* marker, and *P_STE2_-EGFP*	Fukuda et al. [17]
pLY-3GC	*2* µ ori, *LEU2* marker, and *P_STE3_-EGFP*	Fukuda et al. [17]
pHY-PGA	*2* µ ori, *HIS3* marker, and *P_PGK1_-EGFP*	Present study
pHY-Gγ	*2* µ ori, *HIS3* marker, and *P_PGK1_-Gγ*	Present study
pHY-Gγc	*2* µ ori, *HIS3* marker, and *P_PGK1_-Gγ_cyto_*	Present study
pHY-6Gγc	*2* µ ori, *HIS3* marker, and *P_PGK1_-MP6-Gγ_cyto_*	Present study
pHY-6LGγc	*2* µ ori, *HIS3* marker, and *P_PGK1_-MP6L-Gγ_cyto_*	Present study
pHY-10YGγc	*2* µ ori, *HIS3* marker, and *P_PGK1_-MP10Y-Gγ_cyto_*	Present study
pHY-10HiiGγc	*2* µ ori, *HIS3* marker, and *P_PGK1_-MP10Hii-Gγ_cyto_*	Present study
pHY-10HiiiGγc	*2* µ ori, *HIS3* marker, and *P_PGK1_-MP10Hiii-Gγ_cyto_*	Present study
pHY-8YLGγc	*2* µ ori, *HIS3* marker, and *P_PGK1_-MP8YL-Gγ_cyto_*	Present study
pHY-6L2YGγc	*2* µ ori, *HIS3* marker, and *P_PGK1_-MP6L2Y-Gγ_cyto_*	Present study
pHY-G2A-Gγc	*2* µ ori, *HIS3* marker, and *P_PGK1_-G2A-Gγ_cyto_*	Present study
pHY-C3A-Gγc	*2* µ ori, *HIS3* marker, and *P_PGK1_-C3A-Gγ_cyto_*	Present study
pHY-GCA-Gγc	*2* µori, *HIS3* marker, and *P_PGK1_-G2A-C3A-Gγ_cyto_*	Present study
pHY-Gγ-G	*2* µ ori, *HIS3* marker, and *P_PGK1_-Gγ-EGFP*	Present study
pHY-Gγc-G	*2* µ ori, *HIS3* marker, and *P_PGK1_-Gγ_cyto_-EGFP*	Present study
pHY-6Gγc-G	*2* µ ori, *HIS3* marker, and *P_PGK1_-MP6-Gγ_cyto_-EGFP*	Present study
pHY-6LGγc-G	*2* µ ori, *HIS3* marker, and *P_PGK1_-MP6L-Gγ_cyto_-EGFP*	Present study
pHY-10YGγc-G	*2* µ ori, *HIS3* marker, and *P_PGK1_-MP10Y-Gγ_cyto_-EGFP*	Present study
pHY-10HiiGγc-G	*2* µ ori, *HIS3* marker, and *P_PGK1_-MP10Hii-Gγ_cyto_-EGFP*	Present study
pHY-10HiiiGγc-G	*2* µ ori, *HIS3* marker, and *P_PGK1_-MP10Hiii-Gγ_cyto_-EGFP*	Present study
pHY-8YLGγc-G	*2* µ ori, *HIS3* marker, and *P_PGK1_-MP8YL-Gγ_cyto_-EGFP*	Present study
pHY-6L2YGγc-G	*2* µ ori, *HIS3* marker, and *P_PGK1_-MP6L2Y-Gγ_cyto_-EGFP*	Present study
pHY-G2A-GγcG	*2* µ ori, *HIS3* marker, and *P_PGK1_-G2A-Gγ_cyto_-EGFP*	Present study
pHY-C3A-GγcG	*2* µ ori, *HIS3* marker, and *P_PGK1_-C3A-Gγ_cyto_-EGFP*	Present study
pHY-GCA-GγcG	*2* µ ori, *HIS3* marker, and *P_PGK1_-G2A-C3A-Gγ_cyto_-EGFP*	Present study
pUYG-Cre	2 µ ori, *URA3* marker, and *P_GAL1_*-*CRE*	Fukuda et al. [17]

### Construction of Plasmids

The sequences of oligonucleotides used in this study are shown in Table 2. The plasmids shown in Table 1 were made as follows. Using BY4741 genomic DNA as a template, the promoter of the *PGK1* gene was amplified with oligonucleotide pair o1 and o2, and inserted in place of the promoter of the *STE2* gene at the *Sac*II-*Not*I sites of pHY-2GA [17], yielding the plasmid pHY-PGA.

**Table 2:** Sequence of oligonucleotides used to construct plasmids and yeast strains.

Number	Sequence
1	5′-ttttCCGCGGaaagatgccgatttgggcgc-3′
2	5′-aaaaGCGGCCGCgttttatatttgttgtaa-3′
3	5′-ttttGCGGCCGCatgacatcagttcaaaac-3′
4	5′-ccccGGATCCttacataagcgtacaacaaa-3′
5	5′-ccccGGATCCttaaacactatttgagtttgac-3′
6	5′-aaaaGCGGCCGCatggggtgtacagtgagtatgacatcagttcaaaac-3′
7	5′-aaaaGCGGCCGCatggggtgtacagtgagtggtggaggcagtatgacatcagttcaaaac-3′
8	5′-aaaaGCGGCCGCatggggtgtacagtgagtacgcaaacaataatgacatcagttcaaaac-3′
9	5′-aaaaGCGGCCGCatggggtgtacagtgagtgctgaagacaaaatgacatcagttcaaaac-3′
10	5′-aaaaGCGGCCGCatggggtgtacactgagtgctgaagacaaaatgacatcagttcaaaac-3′
11	5′-aaaaGCGGCCGCatggggtgtacagtgagtacgcaaggcagtatgacatcagttcaaaac-3′
12	5′-aaaaGCGGCCGCatggggtgtacagtgagtggtggaacaataatgacatcagttcaaaac-3′
13	5′-atataaaacGCGGCCGCatggcgtgtacagtgagt-3′
14	5′-ccccagtttgGGATCCttaaacactatttgagtttgac-3′
15	5′-atataaaacGCGGCCGCatgggggctacagtgagt-3′
16	5′-atataaaacGCGGCCGCatggcggctacagtgagt-3′
17	5′-ttttGTCGACcataagcgtacaacaaacac-3′
18	5′-ttttGTCGACaacactatttgagtttgaca-3′
19	5′-caaacgttctcaataattctaagaCTCGAGtcgactacgtcgtaaggccg-3′
20	5′-tttttttggattctattactatcaTCTAGAtcgacggtcgaggagaactt-3′
21	5′-atattatatatatatatagg-3′
22	5′-cggccttacgacgtagtcgaCTCGAGtcttagaattattgagaacgtttg-3′
23	5′-aagttctcctcgaccgtcgaTCTAGAtgatagtaatagaatccaaaaaaa-3′
24	5′-ctatgttttggtgtaccgaa-3′
25	5′-aaaaGCGGCCGCatggggtgtacagtgagtNNKNNKacaataatgacatcagttcaaaac-3′

Using BY4741 genomic DNA as a template, each DNA fragment encoding Gγ, Gγ_cyto_, MP6-Gγ_cyto_, MP6L-Gγ_cyto_, MP10Y-Gγ_cyto_, MP10Hii-Gγ_cyto_, MP10Hiii-Gγ_cyto_, MP8YL-Gγ_cyto_ or MP6L2Y-Gγ_cyto_ was amplified with oligonucleotide pair o3 and o4, o3 and o5, o6 and o5, o7 and o5, o8 and o5, o9 and o5, o10 and o5, o11 and o5, or o12 and o5, and inserted in place of the *EGFP* at the *Not*I-*Bam*HI sites of pHY-PGA, yielding the plasmids pHY-Gγ, pHY-Gγc, pHY-6Gγc, pHY-6LGγc, pHY-10YGγc, pHY-10HiiGγc, pHY-10HiiiGγc, pHY-8YLGγc and pHY-6L2YGγc, respectively.

Using plasmid pHY-10YGγc as a template, each DNA fragment encoding G2A-Gγ_cyto_, C3A-Gγ_cyto_ or G2A-C3A-Gγ_cyto_ was amplified with oligonucleotide pair o13 and o14, o15 and o14, or o16 and o14, and inserted in place of the *EGFP* at the *Not*I-*Bam*HI sites of pHY-PGA [17], yielding the plasmids pHY-G2A-Gγc, pHY-C3A-Gγc and pHY-GCA-Gγc, respectively.

For expression analyses, using BY4741 genomic DNA as a template, each DNA fragment encoding Gγ, Gγ_cyto_, MP6-Gγ_cyto_, MP6L-Gγ_cyto_, MP10Y-Gγ_cyto_, MP10Hii-Gγ_cyto_, MP10Hiii-Gγ_cyto_, MP8YL-Gγ_cyto_, MP6L2Y-Gγ_cyto_, G2A-Gγ_cyto_, C3A-Gγ_cyto_ or G2A-C3A-Gγ_cyto_ was amplified with oligonucleotide pair o3 and o17, o3 and o18, o6 and o18, o7 and o18, o8 and o18, o9 and o18, o10 and o18, o11 and o18, or o12 and o18, and inserted into the *Not*I-*Sal*I sites of pHY-PGA, yielding the plasmids pHY-Gγ-G, pHY-Gγc-G, pHY-6Gγc-G, pHY-6LGγc-G, pHY-10YGγc-G, pHY-10HiiGγc-G, pHY-10HiiiGγc-G, pHY-8YLGγc-G, and pHY-6L2YGγc-G, respectively.

Using plasmid pHY-10YGγc as a template, each DNA fragment encoding G2A-Gγ_cyto_, C3A-Gγ_cyto_ or G2A-C3A-Gγ_cyto_ was amplified with oligonucleotide pair o13 and o18, o15 and o18, or o16 and o18, and inserted into the *Not*I-*Sal*I sites of pHY-PGA, yielding the plasmids pHY-G2A-Gγc-G, pHY-C3A-Gγc-G and pHY-GCA-Gγc-G, respectively.

### Construction of Yeast Strains and Yeast Transformation

The plasmid pUYG-Cre [17], which expresses Cre recombinase under the control of the promoter of the *GAL1* gene, was introduced into MCF4741 using the lithium acetate method [18]. To exclude the *kanMX4* gene between the two *loxP* sites, a transformant harboring pUYG-Cre was cultivated in SGal media (containing 6.7% yeast nitrogen base without amino acids; Becton Dickinson and Company, Franklin Lakes, NJ, USA, and 2% galactose) without uracil but containing 20 mg/l histidine, 30 mg/l leucine and 20 mg/l methionine. Then the cells were maintained on YPD media containing 5-fluoroorotic acid (5-FOA) to select *ura3^−^* strains that have lost the plasmid pUYG-Cre, yielding MCF-B1.

Deletion of *STE18* gene by substitution with *LEU2* was carried out as follows. The upstream region of *STE18* was termed *STE*5*’*, and the downstream region *STE3’*. The *LEU2* gene was amplified with oligonucleotide pair o19 and o20, using pLY-3GC [17] as a template. Using genomic DNA from strain BY4741 as the template, *STE*5*’* was amplified with oligonucleotide pair o21 and o22, and *STE*3*’* was amplified with oligonucleotide pair o23 and o24. DNA fragments containing *STE5’*-*LEU2*-*STE3’* were combined using oligonucleotide pair o21 and o24 from the amplified fragments *STE5’*, *LEU2* and *STE3’*, and then used to transform MC-F1 using the lithium acetate method. Transformants were selected on SD plates without leucine (SD – Leu) but containing 20 mg/l histidine, 20 mg/l uracil and 20 mg/l methionine. The resulting strain was named MCF-B1L. Each plasmid shown in Table 1 was introduced into MCF-B1L using the lithium acetate method and the transformants were used for the following analyses.

### Diploid Growth Assay

Evaluation of mating ability was performed as follows. Each engineered yeast strain was cultivated in 1 ml of YPD medium with the mating partner BY4742 at 30°C for 1.5 h setting the initial OD_600_ of each haploid cell at 0.1. After cultivation, yeast cells were harvested, washed, and resuspended in distilled water. Setting the OD_600_ at 1, 0.1 and 0.01, 10 µl of each cell suspension were spotted on an SD plate without methionine and lysine but containing 20 mg/l, histidine, 30 mg/l leucine, and 20 mg/l uracil (SD – Met, Lys plate) for growth selection of diploid cells. After incubation at 30°C for 2 days, we recorded image data of diploid colonies generated on solid medium. For quantitative evaluation, cell suspensions were spread on SD – Met, Lys plates using the appropriate dilution factor for each strain. After incubation at 30°C for 2 days, the colony number was determined for each strain and multiplied by its dilution factor to estimate the number of diploid cells generated in an equivalent volume of 1 ml of cell suspension, with the OD_600_ set at 1.0.

### Fluorescent Reporter Assay

The *EGFP* gene was fused to the C-terminus of *Gγ_cyto_* and used as a fluorescent reporter to indicate expression of Gγ_cyto_-fused hybrid proteins in yeast cells. The cells were incubated in SD – His medium at 30°C for 18 h, harvested and washed with distilled water. The cells were resuspended in 100 µl of distilled water to an optical density of 5.0 at 600 nm (OD_600_ = 5.0). GFP fluorescence intensities were measured on an Infinite 200, fluorescence microplate reader (Tecan Japan Co., Ltd., Kawasaki, Japan). The excitation wavelength was set at 485 nm with the bandwidth of 20 nm and emission wavelength was set at 535 nm with the bandwidth of 25 nm for green fluorescence detection; the gain was set at 50.

### Microscopic Observation

The plasmid pHY-Gγc-G, pHY-6Gγc-G, pHY-6LGγc-G, pHY-10YGγc-G, pHY-10HiiGγc-G, pHY-10HiiiGγc-G, pHY-8YLGγc-G, pHY-6L2YGγc-G, pHY-G2A-Gγc-G, pHY-C3A-Gγc-G or pHY-GCA-Gγc-G was introduced into diploid strain BY4743 (Table 1) to indicate localization of each Gγ_cyto_ hybrid protein in yeast cells. The cells were incubated in SD – His medium at 30°C for 18 h, harvested and washed with distilled water. The localization patterns of GFP-fused Gγ_cyto_ hybrids were observed using a confocal microscopy (Zeiss LSM Pascal 5), using a 63× oil PlanApo objective lens (Zeiss). Images were collected with the same laser power and detector settings using Pascal software version 3.2. To observe green fluorescence, 488 nm laser was used.

### Preparation of Library and Diploid Growth Screening

DNA fragments encoding Met_1_-Gly_2_-Cys_3_-Thr_4_-Val_5_-Ser_6_-Xaa_7_-Xaa_8_-Thr_9_-Ile_10_-Gγ_cyto_ were amplified from plasmid pHY-M10YGγ_cyto_ with the oligonucleotide pair o25 containing tandem NNK codons and o5, and inserted in place of *EGFP* at the *Not*I-*Bam*HI sites of pHY-PGA, yielding a plasmid library. The library was introduced into the MCF-B1L strain and transformants were grown in SD – His medium or plated at 30°C for 2 days to yield an initial yeast library expressing Met_1_-Gly_2_-Cys_3_-Thr_4_-Val_5_-Ser_6_-Xaa_7_-Xaa_8_-Thr_9_-Ile_10_-Gγ_cyto_.

Then the initial yeast library grown in SD – His medium was cultivated in 1 ml of YPD medium with the mating partner BY4742 at 30°C for 1.5 h setting the initial OD_600_ of each haploid cell at 0.1. After cultivation, yeast cells were harvested, washed, and resuspended in distilled water. Cell suspensions were spread on SD – Met, Lys, His plates using the appropriate dilution factor for each strain. After incubation at 30°C for 2 days, plasmids were extracted from 100 randomly picked diploid colonies and sequences corresponding to Xaa_7_–Xaa_8_ were analyzed using the 3500 Genetic Analyzer (Applied Biosystems Inc, CA, USA), together with plasmids extracted from 100 randomly picked colonies from the initial library.

## Results

### Membrane-targeting Ability of the N-terminal Motif Derived from Yeast Gα

**Figure 2:**
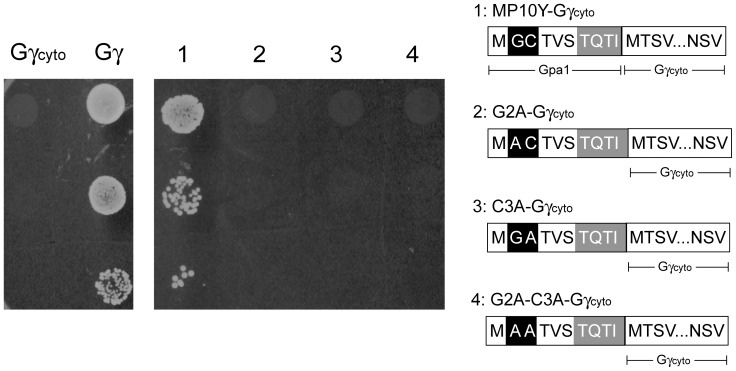
Assessment of the function of N-terminal motifs derived from yeast Gα. The diploid growth assay was used to test the mating ability of yeast cells expressing Gγ_cyto_, intact Gγ, MP10-Gγ_cyto_, G2A-Gγ_cyto_, C3A-Gγ_cyto_ or G2A-C3A-Gγ_cyto_.

Our strategy to investigate protein-lipid association is outlined in Figure 1. The *ste18Δ* strain MCF-B1L (lacking endogenous Gγ) was used as a host strain for yeast one-hybrid GRS. To confirm *STE18* gene deletion and restoration of signal transduction by recruitment of Gγ to the plasma membrane, a diploid growth assay was carried out using MCF-B1L with the plasmid pHY-Gγc, which encodes Gγ_cyto_ (a mutant lacking C-terminal lipidation site), or with the plasmid pHY-Gγ, which encodes wild-type Gγ (Fig. 2). Introduction of Gγ_cyto_ did not result in diploid colonies, consistent with a failure in signaling in the absence of membrane localization. In contrast, introduction of wild-type Gγ, which is expected to localize to the plasma membrane due to the C-terminal lipidations, restored signaling, as evidenced by the production of diploid colonies.

We next attached the N-terminal 10 AA from the N-terminus of Gpa1 (yeast Gα subunit) to Gγ_cyto_ (MP10Y-Gγ_cyto_) in order to create a potential lipidation site receiving myristoylation and palmitoylation. As shown in Figure 2, MP10Y-Gγ_cyto_ successfully restored signaling, resulting in diploid cells, although the number of diploid cells generated from MP10Y-Gγ_cyto_ was lower than from wild-type Gγ. These results suggest that it is possible to evaluate the membrane-targeting ability of additional motifs fused to Gγ_cyto_ as described in Figure 1. We additionally investigated the contribution of myristoylation and palmitoylation to the membrane-targeting ability of MP10Y-Gγ_cyto_. Neither lipidation-defective mutants G2A- (lacking myristoylation-acceptor site), C3A- (lacking palmitoylation-acceptor site) and G2A-C3A-Gγ_cyto_ (lacking both sites) restored the signal transduction at all, suggesting that both lipid modifications are required.

### Contribution of the N-terminal Sequence from Position 7 to 10 to Membrane Targeting

**Figure 3:**
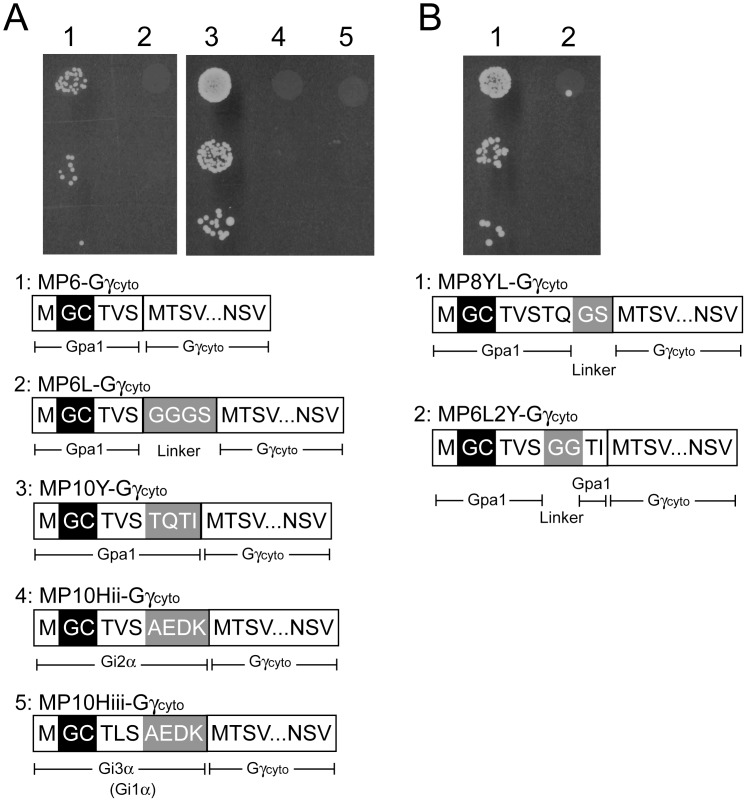
Contribution of N-terminal positions 7 to 10 to the efficiency of dual lipidation. The diploid growth assay was used to test the mating ability of (A) yeast cells expressing MP6-Gγ_cyto_, MP6L-Gγ_cyto_, MP10Y-Gγ_cyto_, MP10Hii-Gγ_cyto_ or MP10Hiii-Gγ_cyto_, and (B) those expressing MP8YL-Gγ_cyto_ or MP6L2Y-Gγ_cyto_.

Yeast Gpa1 and human G_i2_α, receiving both myristoylation and palmitoylation, has the common N-terminal 6 AA (MP6 sequence; Met_1_-Gly_2_-Cys_3_-Thr_4_-Val_5_-Ser_6_). To confirm its function for N-terminal dual lipidations, MP6 sequence was attached to the N-terminus of Gγ_cyto_ (MP6-Gγ_cyto_). As shown in Figure 3A, yeast cells expressing MP6-Gγ_cyto_ generated diploid cells, although the number of diploid cells recovered was lower than what was observed for MP10-Gγ_cyto_ (Fig. 2). Next, an irrelevant linker sequence, Gly-Gly-Gly-Ser, was inserted before Gγ_cyto_ (MP6L-Gγ_cyto_; Fig. 3A). In the diploid growth assay, MP6L-Gγ_cyto_ did not recover signal transduction. These results suggest that the N-terminal 6 AA alone are not sufficient to complete dual lipidation.

To find a sequence that influences N-terminal dual lipid modification, 10 AA from the N-terminus of human G_i2_α or G_i3_α (G_i1_α contains the same 10 AA at the N-terminus as G_i3_α) were attached to Gγ_cyto_, yielding MP10Hii-Gγ_cyto_ or MP10Hiii-Gγ_cyto_. In diploid growth assay, neither Gγ_cyto_ hybrid resulted in diploid cell formation (Fig. 3A). These results are in agreement with past reports that the N-terminal sequence of human G_i2_α and G_i3_α are not substrates of yeast NMT [4]–[5].

To further investigate the contribution of the sequence from position 7 to position 10 to N-terminal dual lipid modifications, two kinds of Gγ_cyto_ hybrids were prepared (Fig. 3B). MP6L2Y-Gγ_cyto_ contains an irrelevant linker sequence (Gly-Gly) at position 7 and 8 of the N-terminal 10 AA sequence derived from Gpa1 and MP8YL-Gγ_cyto_ contains an irrelevant linker sequence (Gly-Ser) at position 9 and 10. In the diploid growth assay, MP8YL-Gγ_cyto_ restored diploid cell formation but MP6L2Y-Gγ_cyto_ resulted in only a few diploid colonies (Fig. 3B). This suggests that in addition to the MP6 sequence, the N-terminal positions 7 and 8 also contribute largely to dual lipid modifications, and that positions 9 and 10 have subtle but definite effects on these modifications.

### Quantitative Assessment of the Membrane-targeting Ability of Gγ_cyto_ Hybrids

**Figure 4:**
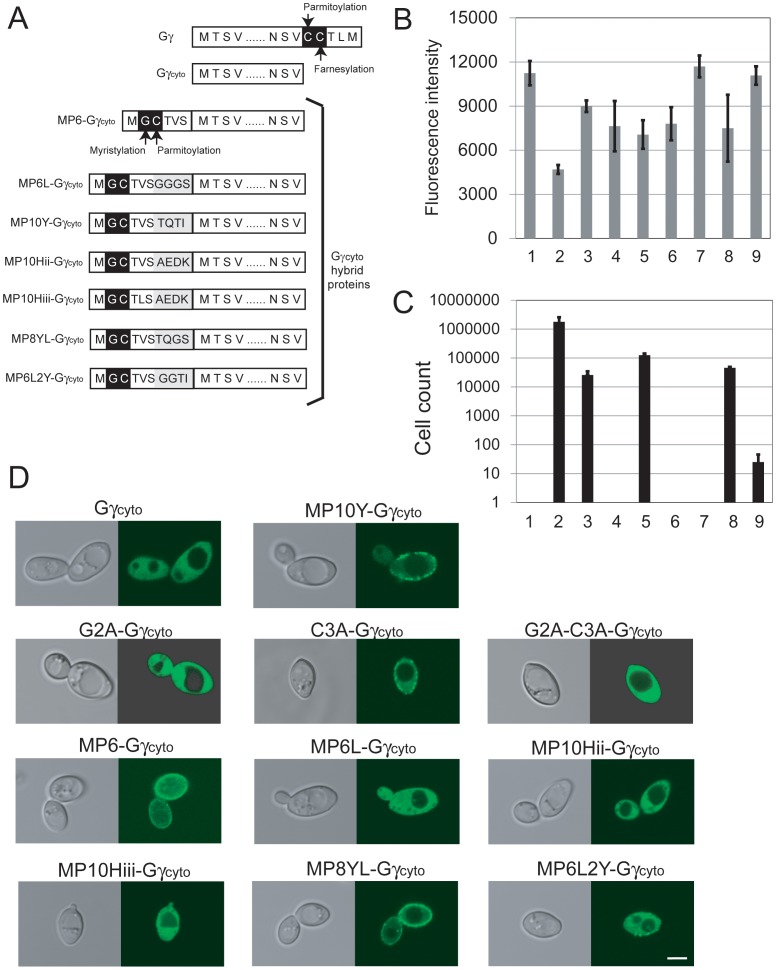
Expression levels and membrane-targeting ability of Gγ_cyto_-hybrids. (A) The N-terminal sequence of Gpa1 (yeast Gα subunit), which confers membrane-targeting ability to the protein. Gpa1 receives dual lipid modification comprised of myristoylation and palmitoylation at N-terminal Gly and Cys residues, respectively, leading to recruitment to the plasma membrane. The membrane-targeting ability of N-terminal sequences derived from several different Gα subunits were evaluated using yeast one-hybrid GRS. (B) A fluorescent reporter assay was used to quantify expression of a *GFP* reporter fused to Gγ_cyto_ hybrids. The standard deviations of three independent experiments are presented. (C) Quantitative cell count following the diploid growth assay to investigate the membrane-targeting ability of Gγ_cyto_ hybrids. Standard deviations of three independent experiments are presented. Gγ_cyto_ (lane 1), Gγ (lane 2), MP6-Gγ_cyto_ (lane 3), MP6L-Gγ_cyto_ (lane 4), MP10Y-Gγ_cyto_ (lane 5), MP10Hii-Gγ_cyto_ (lane 6), MP10Hiii-Gγ_cyto_ (lane 7), MP8YL-Gγ_cyto_ (lane 8), MP6L2Y-Gγ_cyto_ (lane 9). (D) Localization pattern of GFP fused to the C-terminus of Gγ_cyto_ or each Gγ_cyto_ hybrid protein in yeast cells. Scale bar: 5 µm.

To compare expression levels among Gγ_cyto_ hybrids shown in Figure 4A, a GFP reporter was fused to the C-terminus of Gγ_cyto_ or wild-type Gγ. Figure 4B shows the fluorescence intensity of each yeast cell suspension. All Gγ_cyto_ hybrids were successfully synthesized in yeast. Moreover, based on GFP fluorescence, we found that the levels of expression of the hybrid proteins were higher than that observed for wild-type Gγ. We next used the number of colonies generated in the diploid growth assay as a quantitative measure of the membrane-targeting ability of each Gγ_cyto_ hybrid (Fig. 4C). With the exception of wild-type Gγ, MP10Y-Gγ_cyto_ produced the most diploid colonies, suggesting that the N-terminal 10 AA derived from Gpa1 lead to efficient attachment of both myristate and palmitate.

To confirm a direct relationship between membrane-localization of Gγ_cyto_ hybrids and recovery of signal transduction, we observed yeast cells expressing Gγ_cyto_ hybrid-GFP using a confocal microscopy (Fig. 4D). While G2A-Gγ_cyto_, G2A-C3A-Gγ_cyto_, MP6L-Gγ_cyto_, MP10Hii-Gγ_cyto_ and MP10Hiii-Gγ_cyto_ were localized in cytoplasm as well as Gγ_cyto_ that completely lacks membrane-targeting ability, other Gγ_cyto_ hybrids were detected at plasma membrane with different degrees of intensity. The intensity of green fluorescence from the juxtamembrane region, except for C3A-Gγ_cyto_, relatively reflected the efficiency of MP6-Gγ_cyto_, MP10Y-Gγ_cyto_, MP8YL-Gγ_cyto_ and MP6L2Y-Gγ_cyto_ at which signal transduction was restored.

### Exploration of Amino Acid Residue Preference via Screening of a Library Randomized at N-terminal Positions 7 and 8

**Figure 5:**
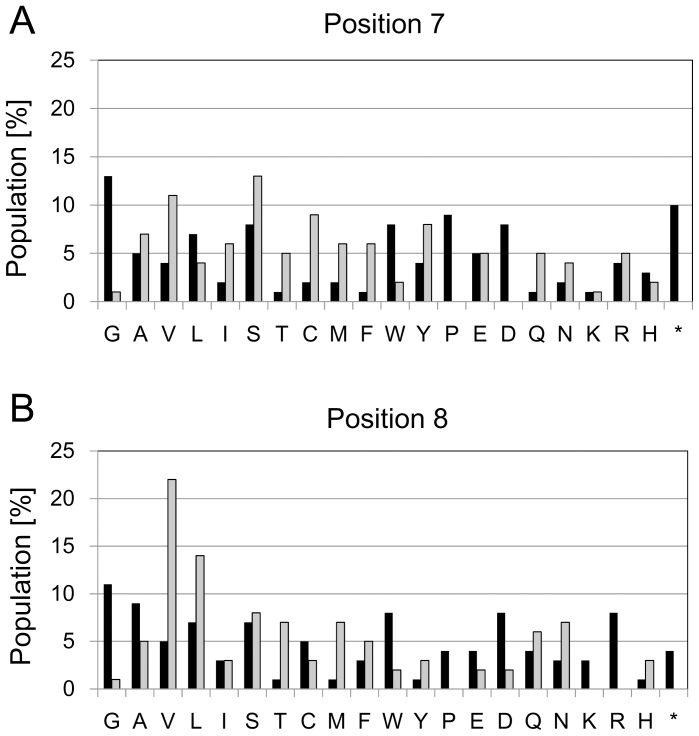
Amino acid residue preferences at positions 7 and 8 for N-terminal dual lipidation. Frequency of each amino acid residue at (A) position 7 and (B) position 8 in plasmids extracted from 100 randomly picked colonies. The symbol * indicates amber stop codon. Black columns, frequencies before screening (initial); gray columns, frequencies after screening (final).

A randomized DNA library encoding Met_1_-Gly_2_-Cys_3_-Thr_4_-Val_5_-Ser_6_-Xaa_7_-Xaa_8_-Thr_9_-Ile_10_-Gγ_cyto_ was prepared by PCR amplification using primers with NNK codons (N represents an equal mixture of A, G, C, and T; K represents an equal mixture of G and T) covering all 20 amino acids, followed by introduction into the MCF-B1L strain (initial yeast library). After dilution and isolation of initial yeast library colonies on solid media, the population of amino acid residues present at positions 7 and 8 was determined by DNA sequencing of plasmids from 100 randomly picked colonies (‘initial’ in Fig. 5).

We next randomly picked 100 colonies grown in the diploid growth assay and determined the frequency of each amino acid residue at positions 7 and 8 (‘final’ in Fig. 5). The ratio of enrichment for each amino acid residue was defined as the value for final population dived by that for initial population (Table 3). Uncharged hydrophilic residues, *i.e.,* Ser, Thr, Asn and Gln, were preferred at positions 7 and 8. No preference was observed for charged residues, *i.e.,* Asp, Glu, Arg, Lys and His, with the exception of a preference for His at position 8. We never observed Asp at position 7, or Arg or Lys at position 8 (ratio of enrichment of 0 in all three cases), suggesting that those residues are incompatible with lipid modification. Cys is known to be a potent acceptor for palmitoylation. In this assay, Cys was enriched at position 7 but not at position 8. Aromatic residues Phe and Tyr, but not Trp (the largest amino acid), were preferred at positions 7 and 8. Among hydrophobic residues, Ala, Val, Ile and Met were preferred at position 7, and Val, Leu and Met were enriched at position 8. Moreover, Gly and Pro were rarely or never seen at either position 7 or position 8.

**Table 3:** Frequency of each amino acid residue at positions 7 and 8 before and after diploid growth screening.

	Position 7			Position 8		
Residue	Pi [%]	Pf [%]	Er	Pi [%]	Pf [%]	Er
G	13	1	0.08	11	1	0.09
A	5	7	1.40	9	5	0.56
V	4	11	2.75	5	22	4.40
L	7	4	0.57	7	14	2.00
I	2	6	3.00	3	3	1.00
S	8	13	1.63	7	8	1.14
T	1	5	5.00	1	7	7.00
C	2	9	4.50	5	3	0.60
M	2	6	3.00	1	7	7.00
F	1	6	6.00	3	5	1.67
W	8	2	0.25	8	2	0.25
Y	4	8	2.00	1	3	3.00
P	9	0	0.00	4	0	0.00
E	5	5	1.00	4	2	0.50
D	8	0	0.00	8	2	0.25
Q	1	5	5.00	4	6	1.50
N	2	4	2.00	3	7	2.33
K	1	1	1.00	3	0	0.00
R	4	5	1.25	8	0	0.00
H	3	2	0.67	1	3	3.00
*	10	0	0.00	4	0	0.00

Pi indicates initial population, Pf indicates final population, Er indicates Enrichment ratio, and symbol * indicates amber stop codon.

### Verification of Amino Acid Residue Preferences Conferring Gγ_cyto_ Membrane-targeting Ability

**Figure 6:**
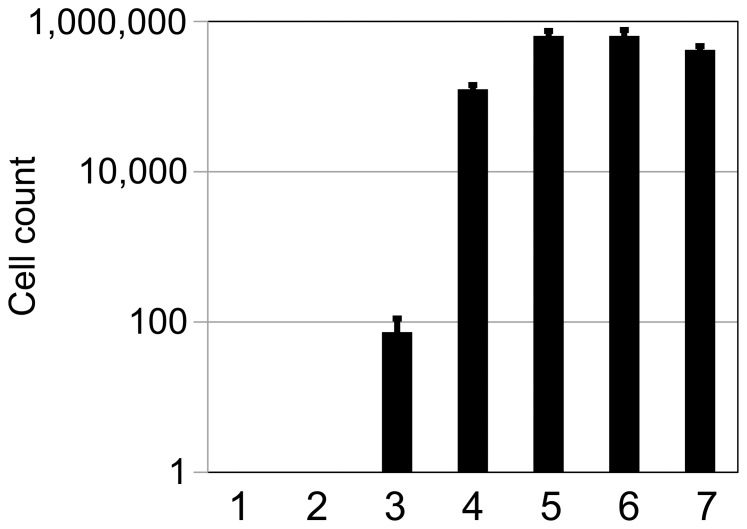
Reliability of predicted amino acid residue preferences. The membrane-targeting ability of each Gγ_cyto_-hybrid was quantitatively measured using the diploid growth assay. Standard deviations of three independent experiments are presented. DW-Gγ_cyto_ (lane 1), GE-Gγ_cyto_ (lane 2), WA-Gγ_cyto_ (lane 3), MP10Y-Gγ_cyto_ (lane 4), TM-Gγ_cyto_ (lane 5), QT-Gγ_cyto_ (lane 6), FN-Gγ_cyto_ (lane 7).

To verify the results of the preference test, we next looked at the membrane-targeting ability of the set of Gγ_cyto_-hybrids listed in Table 4 using the diploid growth assay (Fig. 6). DW-, GE- and WA-Gγ_cyto_ hybrids were used as representatives expected to confer low efficiency of lipid modification (Asp, Gly and Trp were enriched the least at position 7). FN-, QT- and TM-Gγ_cyto_ hybrids were used as representatives expected to confer efficient lipid modification (Phe, Gln and Thr were the most enriched residues at position 7). As shown in Figure 6, DW-, GE- and WA-Gγ_cyto_ rarely generated diploid cells. In contrast, FN-, QT- and TM-Gγ_cyto_ restored signal transduction with a higher efficiency than MP10Y-Gγ_cyto_, which contains the wild-type 10 AA from Gpa1. These results suggest that the amino acid residue biases identified using the yeast one-hybrid GRS (see Figure 5 and Table 3) are reliable predictions of protein activity in cells.

**Table 4:** N-terminal sequences of Gγ_cyto_ hybrids used to verify amino acid residue preferences.

Name	Description
DW-Gγ_cyto_	Met_1_-Gly_2_-Cys_3_-Thr_4_-Val_5_-Ser_6_-Asp_7_-Trp_8_-Thr_9_-Ile_10_-Gγ_cyto_
GE-Gγ_cyto_	Met_1_-Gly_2_-Cys_3_-Thr_4_-Val_5_-Ser_6_-Gly_7_-Glu_8_-Thr_9_-Ile_10_-Gγ_cyto_
WA-Gγ_cyto_	Met_1_-Gly_2_-Cys_3_-Thr_4_-Val_5_-Ser_6_-Trp_7_-Ala_8_-Thr_9_-Ile_10_-Gγ_cyto_
TM-Gγ_cyto_	Met_1_-Gly_2_-Cys_3_-Thr_4_-Val_5_-Ser_6_-Thr_7_-Met_8_-Thr_9_-Ile_10_-Gγ_cyto_
QT-Gγ_cyto_	Met_1_-Gly_2_-Cys_3_-Thr_4_-Val_5_-Ser_6_-Gln_7_-Thr_8_-Thr_9_-Ile_10_-Gγ_cyto_
FN-Gγ_cyto_	Met_1_-Gly_2_-Cys_3_-Thr_4_-Val_5_-Ser_6_-Phe_7_-Asn_8_-Thr_9_-Ile_10_-Gγ_cyto_

## Discussion

The aim of this study was to establish a genetic system, yeast one-hybrid GRS, for analyzing membrane-targeting ability of proteins in a living cell (Fig. 1). Our system is based on the observation that G-protein signaling requires localization of Gγ to the inner leaflet of the plasma membrane [11]. In the current study, we focused the sequence involved in N-terminal dual lipid modifications, *i.e.,* myristoylation and palmitoylation. Protein lipidation is a critical modification that in most cases results in protein trafficking to the membrane. Because the attachment of a single lipid is often not sufficient for membrane targeting, an additional lipidation is typically required [19]. Modification via both myristoylation and palmitoylation at the N-terminus is an example of dual lipidation that can enhance the membrane-targeting ability of a protein.

We succeeded in construction of yeast one-hybrid GRS to investigate lipid-protein associations. Use of diploid growth caused by restoration of G-protein signaling has a potency to explore the sequences responsible for protein lipidations with high-throughput. In our system, both myristoylation and palmitoylation of Gγ_cyto_ hybrids are required to restore the signal transduction (See Fig. 2). As shown in Figure 4, our system can relate eventual diploid cell formation to membrane-targeting ability of proteins with the exception of C3A-Gγ_cyto_. Although C3A-Gγ_cyto_ receiving only myristoylation partially localized at the plasma membrane, it did not restore the signal transduction at all (Fig. 2). It has been reported that G-proteins, G-protein-coupled receptors and other regulators are accumulated in lipid rafts in order to trigger intracellular signal transduction [20]–[22]. Because the combination of myristoylation and palmitoylation functions as a defined lipid raft-targeting signal in protein trafficking [23], C3A-Gγ_cyto_ lacking palmitoylation might have failed to localize in lipid rafts. Therefore, the number of generated diploid cells seems to reflect the amount of dually-lipidated Gγ_cyto_ hybrids in a cell.

N-terminal residues position 7 and 8 play some critical role, but it is unclear if these residues are required for either the myristoylation, palmitoylation, or both as shown in Figure 3. Because it has been reported that Ala_7_-Glu_8_ within MP10Hii-Gγ_cyto_ and MP10Hiii-Gγ_cyto_ were not suitable as substrates of yeast NMT [4]–[5], these positions are probably involved in myristoylation directly. Also these positions are at least indirectly involved in palmitoylation because myristoylation is a prerequisite for palmitoylation [11].

Using yeast one-hybrid GRS, we investigated sequences at position 7 and 8 required for dual lipid modifications which can give membrane-targeting ability to proteins (Fig. 5 and 6). We classified the unsuitable or unacceptable residues for dual lipid modifications into three broad categories. Charged residues are common in N-terminal positions 8 to 10 of human Gα subunits in the G_i_α subfamily [14]. As described above, differences in substrate specificity of NMT between yeast and humans suggest that Glu at position 8 is unacceptable, at least for myristoylation. The second category comprised residues with the highest (Gly) or lowest (Pro) degree of rotational freedom [24], which can have extreme effects on main-chain structure. Finally, Trp, the largest amino acid residue, can be classified as the sole member of a last category of amino acids with a high steric barrier.

In conclusion, we have established a new approach, yeast one-hybrid GRS, to facilitate investigation of protein-lipid associations. Using N-terminal sequences derived from Gα subunits as a model case, we showed that it is possible to test membrane-targeting ability of N-terminal motifs for their ability to receive dual lipid modification, *i.e.,* myristoylation and palmitoylation. Our method approved to be reliable and versatile, and as such, use of the method should help promote further advancement in function analyses of proteins within a living cell.
